# The effect of external ankle support on the kinematics and kinetics of the lower limb during a side step cutting task in netballers

**DOI:** 10.1186/2052-1847-6-42

**Published:** 2014-12-22

**Authors:** Andrew John Greene, Max Christian Stuelcken, Richard Murray Smith, Benedicte Vanwanseele

**Affiliations:** Postgraduate Medical Institute, Faculty of Medical Science, Anglia Ruskin University, Chelmsford, UK; Discipline of Exercise and Sport Science, Faculty of Health Science, The University of Sydney, Sydney, Australia; School of Health and Sport Sciences, Faculty of Science, Health, Education and Engineering, University of the Sunshine Coast, Kragujevac, Queensland Australia; Department of Kinesiology, KU Leuven, Leuven, Belgium; Chair of Health Innovation and Technology, Fontys University of Applied Sciences, Eindhoven, Netherlands

**Keywords:** Netball, Sidestep cutting, Biomechanics, External ankle support, Knee joint loading, Internal valgus moment

## Abstract

**Background:**

Excessive knee valgus moments are considered to be a risk factor for non-contact injuries in female athletes. Knee injuries are highly prevalent in netballers and are significant in terms of cost and disability. The aim of the study was to identify if changes in external ankle support mechanisms effect the range of motion and loading patterns at the ankle and knee joint during a sidestep cutting manoeuvre in high performance netball players.

**Methods:**

Netballers with no previously diagnosed ankle or knee injury (n = 10) were recruited from NSW Institute of Sport netball programme. Kinematic and kinetic data were collected simultaneously using a 3-D Motion Analysis System and a force platform to measure ground reaction forces. Players performed repeated side step cutting manoeuvres whilst wearing a standard netball shoe, the same shoe with a lace-up brace and a high-top shoe.

**Results:**

The brace condition significantly reduced ankle joint ROM in the sagittal plane by 8.9° ± 2.4 when compared to the standard netball shoe (p = 0.013). No other significant changes were seen between conditions for either kinematic or kinetic data. All shoe conditions did however produce knee valgus moments throughout the cutting cycle that were greater than those considered excessive in the previous literature (0.59 Nm/kg-Bwt).

**Conclusions:**

The results show that an external ankle support brace can be used to reduce the ROM at the ankle in the sagittal plane without affecting the loading of the joints of the lower limb. Internal varus moments generated at the knee during the task were however greater than values reported in the literature to classify excessive knee joint moments, regardless of the condition. All netballers exhibited lower extremity patterns and alignments previously associated with increased peak external valgus moments including; increasing hip abduction, peak hip flexion and internal rotation during early contact and high laterally directed ground reaction forces. Increased external valgus knee loads have been strongly linked to the development of non-contact injuries at the knee in female athletes and could highlight a potential mechanism for the development non-contact knee injuries in netballers performing side step cutting tasks.

## Background

Netball is a sport played mainly in commonwealth countries and is one of the most popular team sports in Australia [1]. Netball is a predominantly female sport which places high physical demands upon players, requiring them to perform movements such as jumping, braking, lunging, leaping and hopping [2] often at high speed. As a result of this, injury to the joints of the lower limb, and specifically the ankle and knee joints are highly prevalent. Whilst ankle injuries are reported to be the most prominent site of injury in netballers [3–5], injuries to the knee have the potential to be more serious in terms of impairment and treatment costs [3, 5, 6]. In team sports, up to 70% of injuries to the knee joint, and specifically those injuries affecting the anterior cruciate ligament (ACL) have been shown to be non-contact in nature [7]. Non-contact injuries to the knee typically occur during the landing or stance phase of a high impact task that incorporates sudden deceleration and/or rapid changes in direction [8]. Excessive external knee valgus moments are thought to be a significant factor in placing the female knee at greater risk of ACL injury [9]. Added to this is the finding that female athletes are two to eight times more likely to sustain a non-contact ACL injury when compared to male athletes [7, 10]. This may suggest that female netballers undertaking rapid landing and cutting movements such as the side step cutting manoeuvre could be highly susceptible to injuries at the knee.

The high risk of injury at the ankle in netball is one that has been given the most attention both by researchers and athletes. External ankle supports (prophylactic ankle brace; high top shoes) are commonly used in an attempt to protect the ankle joint or to prevent further injury [11] with their most important function being to ensure the necessary stability to avoid inversion injuries [12]. Ankle braces have been shown to be effective in restricting frontal plane motion at the ankle in a netball specific landing tasks [13] and have been shown to significantly reduce the occurrence of ankle sprains in athletes and particularly those with a history of ankle injury [14, 15], although not specifically in netballers. However, some research has suggested that restricting the motion at the ankle may alter the loading at the knee joint. Restricting the ankle motion in the frontal plane using prophylactic ankle braces [16] and custom foot orthotics [17] has been linked to increased peak external rotation moments at the knee joint during vertical landing [16] and running tasks [17], which may have the potential to contribute to the development of knee injuries through altered knee loading [9, 18]. Whilst ankle braces have been shown to restrict frontal plane ankle motion during a netball specific landing task without altering the mechanics at the knee [13], the effect of ankle bracing on side step cutting manoeuvres has not been examined. The literature examining the effect of high top shoes in preventing ankle sprains has been inconclusive as to whether high top shoes have a stabilising effect at the ankle and are able to restrict ankle inversion [19]. There have however been reports that high top shoes, whilst not restricting the ROM at the ankle, increased plantar flexion moments at the ankle, internal rotation moments at the knee and the ROM at the knee during a single leg netball landing whist receiving a pass [13]. Studies have also showed that wearing high top shoes in certain ankle strain situations brought about delayed muscular pre-activation timing, decreased amplitudes of muscle activity and changed proprioceptive feedback, which may have a detrimental effect on establishing and maintaining functional ankle joint stability [19].

A demanding and dynamic movement such as a side step-cut, which requires athletes to change their direction of motion after landing and has been implicated in the development of knee injuries [9], may produce changes in the interaction of the joints of the lower limb with different external ankle support mechanisms. Potential restriction of the ankle motion in the frontal plane from an external ankle support may alter the mechanics at the knee joint in female netballers undertaking this task. The primary objective therefore was to quantify and compare the effect of different ankle support conditions: a standard netball shoe, a standard netball shoe with a supportive ankle brace and a high-top shoe on the ankle joint movement and loading during a side step cutting task. We hypothesised that the external brace and the high-top shoes would restrict the peak ankle joint angles, range of motion (ROM) and position throughout the contact phase of the side step cut. The secondary aim was to examine ankle and knee joint moments during the side step cutting task in the different ankle support conditions. We hypothesised that the brace and the high-top shoes would increase knee joint moments compared to the standard shoes throughout the contact phase of the side step, as a result of restriction of the motion at the ankle.

## Methods

Ten female netballers (mean age, 18.3 ± 1.9 years; height, 178.1 ± 4.0 cm; mass, 69.9 ± 8.5 kg) elected to participate in this part of the study. Each player provided written consent prior to commencement. For those players under the age of 18, parental consent was also obtained. Initially, 44 players from the New South Wales Institute of Sport (NSWIS) netball program completed a self-administered questionnaire which sought information about their experiences with knee and ankle problems. Nineteen players were excluded from the study because they satisfied one or more of the exclusion criteria: (1) a history of knee or ankle surgery; (2) knee or ankle pain in the previous six months that required consultation with a medical practitioner and/or caused a formal netball training session or game to be missed; or (3) current knee or ankle pain or instability that would have prevented performance of the side step cutting task at the required intensity. Of the 25 players that met the inclusion criteria, many were unavailable to participate in the study due to; travel distance, representative netball, other commitments or injuries sustained between completing the screening questionnaire and the time of testing. Players were assessed in an indoor biomechanics laboratory using a protocol that was approved by the Human Research Ethics Committee at the University of Sydney.

A three-dimensional kinematic analysis was performed to track the position of all segments of the right lower limb (pelvis, thigh, shank, rear foot and fore foot, respectively) in space. The data were collected at 200Hz using 14-camera 3-D motion analysis system (Cortex, Motion Analysis Corporation, Santa Rosa, CA, USA). Additionally, one Kistler force plate (Kistler Instruments AG, Winterhur, Switzerland) sampling at 1000 Hz was used to simultaneously measure ground reaction forces. Each subject had twenty-one reflective surface markers attached to specific anatomical landmarks on the pelvis, thigh, shank, calcaneous and shoe to calculate three dimensional kinematic data [13].

Motion at the ankle joint was calculated using a previously described model [20], in which the ankle joint has three degrees of freedom. The multi-segment foot model is based on one used previously in the literature with moderate to high inter session reliability [21, 22]. For all shoe conditions, motion of the rear foot segment was defined by a detachable wand triad marker which was attached directly to the calcaneous. This has been shown to be a valid and reliable method of obtaining in-shoe motion [23]. Wand-based markers are commonly used to measure three-dimensional rear foot kinematics [24]. The wand triad markers extended through a 16 mm diameter hole in the heel counter of the shoe. The use of the detachable rear foot wand cluster ensured marker placement was not altered between conditions, as the base for the markers remained in place during the data collection process. Forefoot motion was tracked by placing reflective markers on the outer of the shoe’s upper [25, 26]. For the ankle brace condition, markers to track the medial and lateral malleolus were palpated and attached to the surface of the ankle brace, so as not to alter the integrity of the ankle brace.

The movement pattern assessed was a side step cutting task during which each player was instructed to use a 5 m straight line approach to the landing area at a self-selected, match appropriate speed. This was calculated using the horizontal velocity of the sacrum marker in the five frames prior to heel contact. All players were required to land on the ground embedded and level force platform and sidestep cut off the right leg at a cutting angle of approximately 45° towards a designated marked location [27]. Players were allowed as many practice trials as necessary to become familiar with the procedures and testing environment and all players identified as right handed/footed. Once data collection commenced players were required to complete 8–10 successful trials. A trial was considered successful if it satisfied the requirements of the task and the right foot landed within the border of the force plate. Players performed this movement with a standard netball shoe (Ignite3, ASICS) (standard condition), the same netball shoe with a lace-up brace (E-Professional) (brace condition) and a high-top shoe (Jordan, Nike) (high-top condition). The order of the conditions was randomized.

Kinematic and kinetic data were processed using Visual3D software (C-motion, Rockville, MD, USA). The lower extremity segments were modeled as a frustra of right cones while the pelvis was modeled as a cylinder. Anthropometric data was used based on [28]. Internal moments were calculated at the proximal end of the distal segment of each joint. The local coordinate systems of the pelvis, thigh, leg, rear-foot and fore-foot were derived from the standing reference position in which participants stood in a relaxed stance with both feet aligned with the laboratory X axis. Players adopted this reference position prior to undertaking the side step cut for each condition. Coordinate data were low-pass filtered using a fourth-order Butterworth filter with a 6–15 Hz cutoff frequency. Ground reaction force data were low-pass filtered using a fourth-order Butterworth filter with a 20 Hz cut-off frequency. Six degrees-of-freedom for each segment were determined from the segment’s set of reflective markers. Subsequently, lower extremity 3-D joint angles were calculated using a XYZ Cardan rotation sequence.

All data were time-normalized to 100% of the cut cycle and all players contacted the ground with their right (dominant) foot. The cut cycle was defined as the period from initial contact of the right foot (0%) to the toe off of the right foot, as determined by the vertical ground reaction forces with a threshold of 20 N. Four trials per subject per condition were analysed. Discrete variables (peak joint angles, joint range of motion, peak joint moments, peak ground reaction forces) were extracted from each individual trial and averaged for each player. All trials were time normalized across stance and averaged for each player. The individual mean curves were then averaged across conditions to produce ensemble curves.

Statistical analyses were undertaken in SPSS 21.0 (IBM SPSS Statistics for Windows, Armonk, NY, USA). For the primary discrete variables of the footwear conditions: Standard shoe vs Brace condition vs High Top Shoe, a repeated measures analysis of variance was undertaken for the ankle and knee joint range of motion, peak joint moments and peak ground reaction forces. A Bonferroni adjustment was applied to each condition to test significant differences between footwear conditions. All *p* values of less than 0.05 were considered statistically significant. Data were expressed as mean and standard deviation.

## Results

No significant differences existed between the approach velocities in each of the three conditions (Standard: 3.2 m.s^-1^ ± 0.4; Brace: 3.3 m.s^-1^ ± 0.4; High Top: 3.3 m.s^-1^ ± 0.4). No significant difference existed for foot progression angle at flat foot between conditions, with all conditions landing within 1° of the zero alignment, as defined by the standing reference trial (Standard: 0.1° ± 1.6; Brace: 0.8° ± 1.5; High Top: 0.1° ± 1.9). The brace condition was shown to significantly reduce the range of motion (ROM) at the ankle in the sagittal plane when compared to the standard shoe condition (39.7° ± 8.4° vs. 48.6° ± 10.6°, p = 0.013) (Table 1). There was no significant effect of the brace or high top condition at the ankle or knee in any of the other planes of motion (Table 1). No significant differences existed at either the ankle or the knee joint for the peak joint moments (Table 2) or for the peak ground reaction forces (Table 3) between the different conditions.

**Figure 1:**
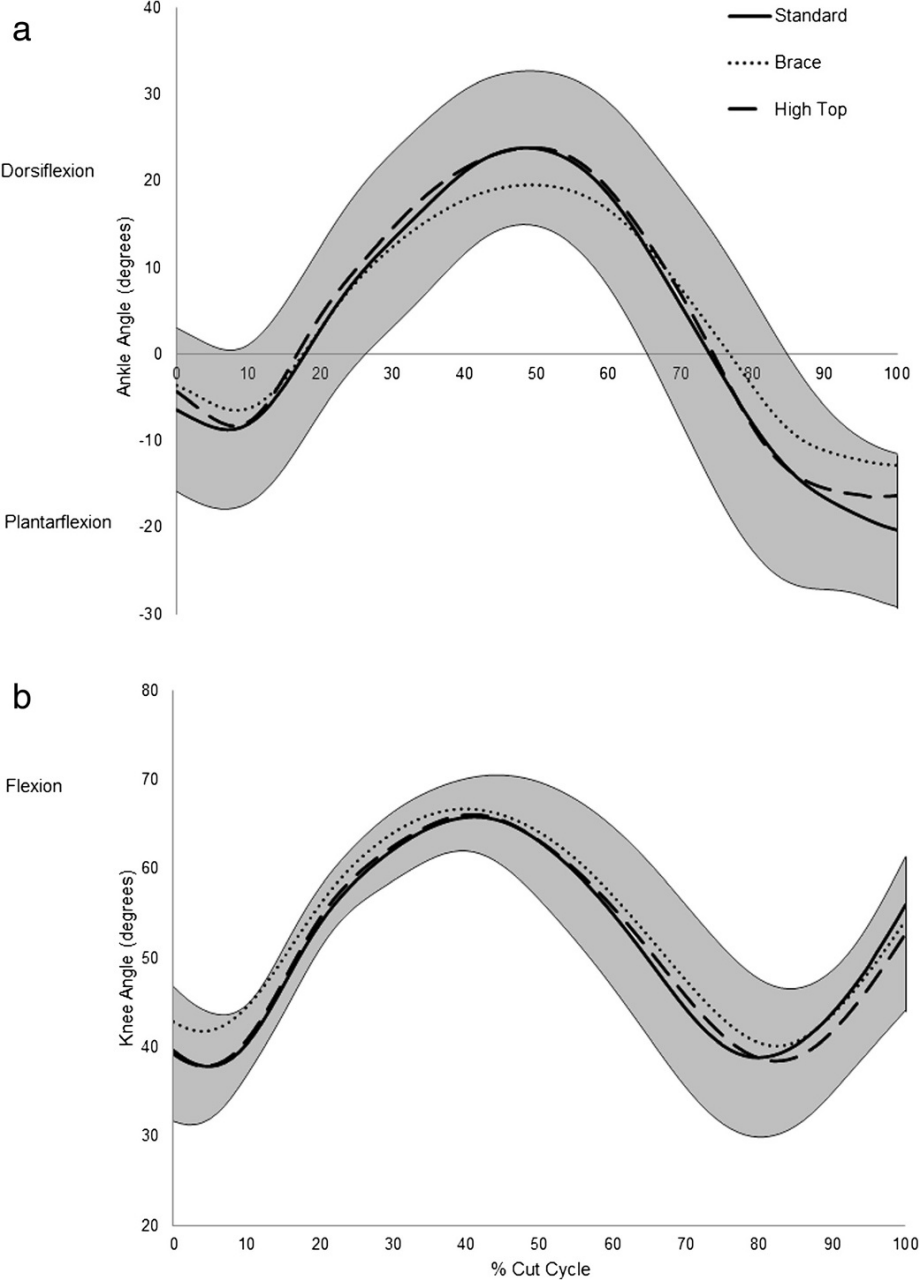
**Sagittal plane angular displacement at the ankle and knee joint.** Mean ensemble curves for **(a)** the sagittal plane ankle angular displacement and **(b)** the sagittal plane knee angular displacement during the cut cycle. The shaded area shows the 95% confidence interval of the mean curve for the standard shoe condition. Dorsiflexion and Flexion are depicted at positive in the figure.

Figure 1 shows the ensemble curves for the sagittal plane kinematics at the ankle and the knee for all conditions. At initial contact, the ankle is slightly plantar flexed and undergoes a slight increase in plantar flexion through the initial loading phase. This occurs with a relatively static knee angle through the first 10% of the phase. The ankle and knee joints simultaneously undergo increases in dorsi flexion and flexion respectively through the next 30% of the phase. The knee reaches peak flexion at approximately 40% of landing after which it undergoes extension through to approximately 80% of ground contact. Ankle dorsi flexion continues to increase until approximately 50%, after which the ankle plantar flexes through to toe off at 100%. The knee once again flexes from approximately 80% of the phase through to toe off.

**Figure 2:**
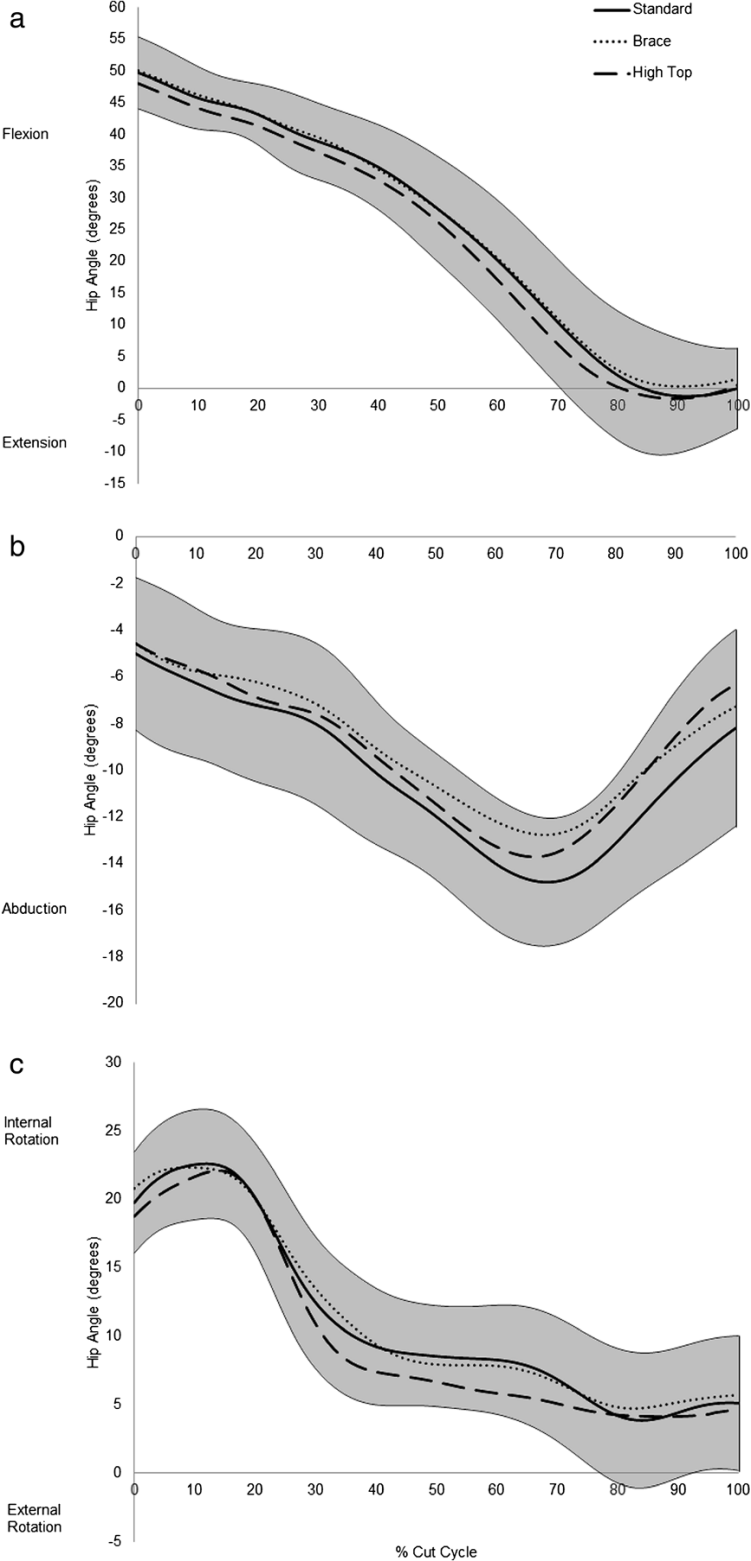
**Sagittal, Frontal and Transverse place angular displacement at the hip joint.** Mean ensemble curves for **(a)** the sagittal plane hip angular displacement **(b)** the frontal plane hip angular displacement and **(c)** the frontal plane hip angular displacement the during the cut cycle. The shaded area shows the 95% confidence interval of the mean curve for the standard shoe condition. Flexion, Adduction and Internal Rotation are depicted as positive in the figure.

Figure 2 shows the kinematics of the hip joint throughout the landing phase of the side step cut. The hip is relatively flexed at initial ground contact and undergoes continual extension throughout the entire phase. Hip extension plateaus at approximately 85% of the landing through to toe off at 100%. In the fontal plane, the hip is abducted at ground contact which continues to rise through to approximately 65-70% of the landing phase, after which the hip adducts through to toe off. The hip is internally rotated at initial contact and this increases slightly through the initial contact phase, up to approximately 15% of the phase. The hip then externally rotates through the remainder of the landing, rapidly from 15-35% of the phase, and then more gradually though to toe off.

**Table 1:** **Range of motion at the ankle and knee joint for all ankle support conditions**

	Standard	Brace	High Top
Ankle Sagittal ROM	48.6 ± 10.6	39.7 ± 8.4*	45.8 ± 6.5
Ankle Frontal ROM	13.5 ± 4.6	13.5 ± 4.4	14.7 ± 6.4
Ankle Transverse ROM	16.1 ± 9.1	14.1 ± 7.4	17.9 ± 5.1
Knee Sagittal ROM	41.1 ± 6.5	38.9 ± 9.1	39.2 ± 7.2
Knee Frontal ROM	8.6 ± 2.8	8.5 ± 3.6	8.4 ± 2.1
Knee Transverse ROM	17.5 ± 4.2	17.6 ± 5.4	17.9 ± 3.3

Values are presented in degrees (°) as mean ± SD. *Significant difference between the Standard shoe condition and the Standard shoe with ankle brace condition (p ≤ 0.05).

**Table 2:** **Peak joint moments at the ankle and knee joint for all ankle support conditions**

	Standard	Brace	High Top
Ankle Flexor Moment	0.04 ± 0.01	0.04 ± 0.02	0.04 ± 0.01
Ankle Extensor Moment	0.07 ± 0.02	0.06 ± 0.02	0.07 ± 0.02
Ankle Inversion Moment	0.01 ± 0.01	0.01 ± 0.01	0.01 ± 0.01
Ankle Eversion Moment	0.01 ± 0.01	0.01 ± 0.01	0.01 ± 0.01
Ankle Adduction Moment	0.03 ± 0.01	0.02 ± 0.01	0.02 ± 0.01
Ankle Abduction Moment	0.03 ± 0.02	0.02 ± 0.02	0.01 ± 0.01
Knee Flexion Moment	3.81 ± 0.42	3.77 ± 0.57	3.91 ± 0.38
Knee Extensor Moment	1.66 ± 0.39	1.58 ± 0.46	1.70 ± 0.32
Knee Varus Moment	0.78 ± 0.29	0.80 ± 0.40	0.87 ± 0.34
Knee Valgus Moment	0.44 ± 0.27	0.36 ± 0.25	0.48 ± 0.29
Knee Internal Rotation Moment	0.34 ± 0.17	0.32 ± 0.13	0.36 ± 0.14
Knee External Rotation Moment	0.37 ± 0.17	0.40 ± 0.18	0.42 ± 0.16

Values are presented in Nm / kg.Bwt as mean ± SD. Positive moments are determined at the ankle as: dorsiflexion, inversion and adduction; and at the knee as: flexion, adduction (varus) and internal rotation.

**Table 3:** **Peak ground reaction forces for all ankle support conditions**

	Standard	Brace	High Top
Vertical GRF	23.1 ± 0.8	23.3 ± 2.7	22.9 ± 2.2
Medial GRF	9.9 ± 0.7	10.1 ± 1.6	9.7 ± 1.4
Breaking GRF	-7.0 ± 2.8	-6.9 ± 2.6	-6.8 ± 2.3
Propulsive GRF	1.6 ± 0.6	1.8 ± 0.3	1.7 ± 0.5

Values are presented in degrees N / kg.Bwt as mean ± SD. Breaking (posterior) GRF is presented at negative as it acts in the opposite to that of the direction of motion.

## Discussion

The brace condition significantly reduced ankle joint range of motion in the sagittal plane by 8.9° ± 2.4° throughout ground contact of the side step cut, when compared to the standard netball shoe. These results differ from previous research that has investigated the effect of ankle bracing in a netball specific landing task [13] in that no restriction of motion in the frontal plane at the ankle was observed in the current study. Restriction of ankle ROM in the current study came without any changes in the ROM at the knee joint, or any increases in the loading at the ankle or knee joint. This is not entirely surprising however, as previous studies [16] have linked frontal plane ROM restriction at the ankle using an external ankle support with increased external rotation moments at the knee, which were not demonstrated in this study. As such, it can be suggested that an external ankle support brace can be used successfully to stabilise the ankle joint and limit range on motion in the sagittal plane during a side step cutting task without increasing the potential for knee injury due to altered mechanics and loading of the knee joint. No significant restrictions were seen for the ankle ROM in the high top shoe condition. It can therefore be suggested that despite observing some minor restriction in the sagittal plane range of motion at the ankle (Table 1), the use of a high top shoe in an attempt to stabilise and restrict the ankle ROM during a side step cutting task is not as effective as an external ankle brace. Unlike in previous studies that examined the effect of external ankle support during a netball specific landing task [13], no alterations to the plantar flexion moments at the ankle, internal rotation moments at the knee or the ROM at the knee were seen in the high top condition. As previously mentioned, it might have been expected that the ankle brace condition would have brought about restrictions in the frontal plane ROM at the ankle throughout the task, and in the ankle inversion eversion angle at initial contact, however this did not occur (data for inversion eversion angle at initial contact not shown). It has been reported in the literature that the most important function of ankle braces is to ensure the necessary stability immediately prior to landing to avoid lateral ankle injuries, however this was assessed in vertical landing tasks where the ankle was loaded on a 30° tilting platform [12]. Previous studies that have found restrictions in the frontal plane ROM in external brace conditions have done so in tasks where the ankle was passively forced into internal or external rotation [29, 30] or where a task involved the stabilisation of the ankle during the active slowing of a forward landing [13]. In the side step cutting task performed in the current study, the athlete is not trying to actively slow down her landing at contact in preparation for a rapid stop as in a catch and land task [13] and is maintaining forward movement which will then be transferred and used to facilitate an effective cutting movement. Otago [1] reported that run-on landing conditions (similar to the side step cut), as opposed to landing conditions that required a rapid deceleration or stop, were the least stressful on the body. As there is less demand on the ankle joint to actively brake and slow down the movement throughout landing during the side step cutting task as opposed to a land and stop task [13], it may therefore be the case that in the side step cutting task, the ankle joint is not subjected to frontal plane loads which require the ankle brace to provide stability and ROM restriction in the frontal plane. It is not entirely evident from the results of this study whether the restriction of the ankle ROM in the sagittal plane in the brace condition is beneficial to the athlete, especially as no significant differences were seen in the joint moments or ground reaction forces experienced by the athletes. These results may however be of benefit to those athletes suffering from chronic ankle instability or recurrent ankle sprains, who may require joint range of motion limitation to provide a greater feeling of stability at the ankle joint, whilst not altering the action of the other joints of the lower limb.

No significant differences existed between conditions for the motion and loading at the knee joint throughout the task. However, all shoe conditions produced internal knee varus moments throughout ground contact (Table 2) that were larger than the external valgus moments considered to be excessive during a side step cutting task in previous literature [9]. The data reported in the current study depicts the internal varus moment at the knee, whereas the previously presented literature [9] shows the external valgus moment at the knee. Due to the different conventions used, these two variables depict comparable loading in the frontal plane on the medial side of the knee. Irrespective of the adopted convention (internal or external joint moment), studies in general report consistent joint moment profiles for the sagittal and frontal planes during able-bodied adult gait [31]. Nine of the ten netballers tested produced internal knee varus moments for at least one of the shoe conditions of a magnitude greater than 0.59 Nm/kg-Bwt, the value used in the study by Sigward and Powers [9] to group athletes that were identified as exhibiting excessive external valgus moment at the knee joint during a side step cut. Whilst the values in the current paper (Standard: 0.78 ± 0.3 Nm/kg-Bwt; Brace: 0.80 ± 0.4 Nm/kg-Bwt; High Top: 0.87 ± 0.34 Nm/kg-Bwt) are not as large as those reported by Sigward and Powers [9] (1.2 ± 0.4 Nm/kg-Bwt), they are still much larger than the 0.59 Nm/kg-Bwt used to define excessive external knee valgus moments, and the external knee valgus values of 0.62 ± 0.2 Nm/kg-Bwt reported by McLean et al. [32] in females during side stepping tasks. The reason for the reduced knee valgus moments in the current study can more than likely be attributed to the reduced approach velocity of the athletes (3.3 ± 0.07 m.s^-1^) as compared to the increased approach velocity (5.1 ± 0.4 m.s^-1^) used previously in other studies [9]. In the context of a netball population however, this would more than likely be an accurate representation of the speeds at which players would carry out such tasks, due to the restrictive court conditions and the relatively short duration of sprint activities that occur in netball games [33].

External knee valgus loading has been reported to be the predominant mechanism of non-contact injury to the ACL during the side step cutting task, and this has been shown to be elevated in female athletes [9, 27, 34]. It is thought that poor or altered neuromuscular control of the knee during sidestep cutting could potentially expose the knee to dangerous combinations of knee joint loading [8, 34, 35]. Increased peak external valgus moments have also been correlated with a number of kinematic actions at the hip which appear to act to increase the valgus load on the knee joint [32]. Athletes exhibiting excessive external valgus knee moments [9] demonstrated increased internal rotation and abduction at the hip and greater laterally directed GRF’s during the side step. Kipp et al. [36] reported that less overall hip flexion throughout the side step cut task had an important role with respect to controlling the frontal and transverse plane loading of the knee, and specifically that reduced hip flexion acted to increase the peak internal knee rotation moments, which are considered to be important dynamic loading mechanisms of the knee [8]. It can be seen from Figure 2 that the netballers assessed in the current study demonstrate a number of these lower extremity patterns and alignments, with continual hip extension and hip abduction throughout the task, initial internal rotation with a rapid shift into external rotation of the hip, as well as high laterally directed GRF’s (Table 3). This may therefore indicate that the netballers tested in the current study are demonstrating kinematic actions at the hip joint which may contribute to the elevated internal valgus moments at the knee joint. A possible mechanism for these actions at the hip would be to facilitate the change in direction during the cutting task, which requires the rotation of the body towards the direction of the cut and the lateral translation of the centre of mass to this direction [9]. Hip internal rotation at initial contact would act to rotate the landing limb towards the direction of motion of the cut. The rapid hip external rotation after landing would suggest that the body is being rotated to the direction of the cut whilst the foot is still planted on the ground. The continual hip extension through the contact phase suggests a more upright body position through the landing, which is similar to previous findings [36] and may therefore increase the demands on the knee joint throughout ground contact. Large laterally directed ground reaction forces as the athlete executes the cutting aspect of the manoeuvre, coupled with the orientation of the body towards the direction of the movement and the increase in hip abduction could act to increase the loading on the medial aspect of the knee.

Given the relatively high incidence and potential severity of knee injuries in netballers, it is important to identify the underlying mechanisms that increase the internal varus/external valgus knee loading, and subsequently the potential risks of injury. The identification of risk factors and the development of prevention strategies may have widespread health and economic implications [34]. It has been suggested that increased hip internal rotation and/or flexion at initial contact may compromise the ability of the medial muscle groups to adequately support the resultant knee valgus loading, and that increased neuromuscular control during sidestepping may reduce the likelihood of ACL injury via valgus load in females [32]. Poor and/or altered neuromuscular control during side step cutting tasks has been suggested as a major contributor to the production of potentially hazardous knee joint loading combinations that place the ACL at risk [8, 34]. Sigward and Powers [9] reported that athletes that displayed normal frontal plane moments during the side step cutting task maintained a more neutral alignment with the centre of pressure of the ground reaction forces closer to the centre of mass throughout the movement. They suggested that instructions of body alignments with the goal of maintaining a more vertical tibia and reduced medio-lateral forces through landing should be included in injury prevention training. This idea for providing postural alignment training to prevent injury may be particularly valid in a netball population, who in previous studies [37] have been shown to have difficulty in consistently aligning the knee and foot during single leg landing tasks. An investigation of netballers that demonstrate both normal and excessive ranges of internal varus or external valgus knee loading is necessary to determine if the side step cutting techniques undertaken by the groups significantly differed, and to see whether the actions at the hip, which have been previously linked to increased external valgus knee loading were present or active in netballers exhibiting reduced internal varus knee moments. Since these actions at the hip throughout the landing have been linked to potential neuromuscular weaknesses in the athletes with elevated valgus knee loads, it would also be advantageous to undertake further work to see whether neuromuscular training interventions to target improvements in the strength of the hip musculature may be beneficial for netballers.

There were a number of limitations of the current study. The sample size of netballers tested was small, which may impact upon the power of our findings and the extent to which the findings can be generalised. Whilst netballers were recruited from the same source to maintain a homogenous sample, access to and the availability of high performance netballers was limited. The current study calculated the internal joint moments at the joints to describe the loads being applied to joints throughout the side step cut. Previous studies [9, 32, 35] have however reported external joint moments acting throughout the side step cutting manoeuvre. Whilst it is suggested that regardless of the convention, internal and external moments are comparable in able bodied gait [31], differences in the methods used to calculate the moments including marker sets, reference frames and joint expression could not be standardised and so should be considered when comparing and reviewing the results. This being said, all moments have been displayed with the same units of Nm/kg-Bwt with the purpose of the results being used to provide some context to suggest possible mechanisms of knee injury in female netballers during a side step task. The velocity of the side step cut in the current study was much slower than those studies that reported external valgus knee loads side step cutting tasks [9, 32] which could potentially have limited the findings of the current study. The approach velocity demonstrated by the netballers in the current study is however close to the 4 m.s^-1^ that has been recommended as the standardised value of approach speed to be used when examining side step cutting tasks in female athletes [38]. All netballers were asked to perform the task at a self-selected game related speed, and as mentioned previously, the velocity of the task in the current study does not seem unreasonable for a sample of netballers given the demands of the game. The side step cutting tasks in the current study was also anticipated by the players, and so they had the opportunity to prepare for the task prior to carrying it out. Studies [36, 39, 40] have suggested that loads are increased when the task is unanticipated in nature, so this needs to be taken into account when evaluating the findings of the current study. Whilst it would be beneficial and interesting to look at unanticipated side step tasks in the netball cohort, in a netball game, athletes would undertake a number of side step cuts where they were anticipating making the move in order to run into space to receive a pass.

## Conclusions

The result show that an external ankle support brace can be used to successfully reduce the sagittal plane ROM at the ankle during a side step cutting task without having any effects upon the loading of the joints of the lower limb. There were no changes in the frontal plane ROM at the ankle between the brace and the other shoe conditions. The data does however show that netballers demonstrated high internal varus moments of the knee joint during the side step cutting task regardless of the external ankle support mechanism. The internal varus loading of the knee in the current study was greater than the external valgus loading values reported by previous studies [9] to highlight athletes that produced valgus knee loads that were classed as excessive. These findings may suggest that the side step cut places greater loads on the knee joint throughout the task with a reduced need to stabilise the ankle joint laterally. Increased external valgus knee loads have been strongly linked to the development of non-contact injuries at the knee in female athletes, and so the results of the current study may highlight a potential mechanism for the development of non-contact injuries at the knee joint in netballers performing side step cutting tasks.

## Abbreviations

ACL: Anterior Cruciate Ligament
ROM: Range of Motion.
